# The Effect of Emotional Self‐Regulation Counseling on Anxiety and Fear of Childbirth in First‐Time Pregnant Women: A Clinical Trial Study

**DOI:** 10.1002/hsr2.71781

**Published:** 2026-02-05

**Authors:** Negar Masoumi, Farideh Kazemi, Azita Tiznobaik, Mohammad Ahmadpanah

**Affiliations:** ^1^ Department of Midwifery and reproductive Health, School of Nursing and Midwifery Hamadan University of Medical Sciences Hamadan Iran; ^2^ Mother and Child Care Research Center, Institute of Health Sciences and Technologies Hamadan University of Medical Sciences Hamadan Iran; ^3^ Behavioral Disorders and Substance Abuse Research Center, Institute of Neuroscience and Mental Health Hamadan University of Medical Sciences Hamadan Iran; ^4^ Department of Psychiatry, School of Medicine Hamadan University of Medical Sciences Hamadan Iran

**Keywords:** emotion regulation, fear of childbirth, Gross's model, midwife‐led intervention, pregnancy‐related anxiety, primiparous women, randomized controlled trial

## Abstract

**Background:**

Pregnancy‐related anxiety and fear of childbirth are common in primiparous women and contribute to high cesarean section rates. Few interventions specifically target emotion regulation skills.

**Objective:**

To evaluate the efficacy of a brief, midwife‐led, group‐based counseling program grounded in Gross's emotion regulation model in reducing pregnancy‐related anxiety and fear of childbirth.

**Methods:**

Pragmatic randomized controlled trial in Hamadan, Iran, 2023. Seventy primiparous women (28–33 weeks gestation) were allocated (1:1) to 6 weekly emotion regulation counseling sessions or routine prenatal care. Primary outcomes were post‐intervention scores on the Persian Pregnancy‐Related Anxiety Questionnaire (PRAQ) and Childbirth Attitudes Questionnaire (CAQ), analyzed by ANCOVA adjusting for baseline scores.

**Results:**

Adjusted mean PRAQ scores were 36.65 (SE 2.74) vs. 66.41 (SE 2.74) (adjusted difference –29.76, 95% CI −37.28 to −22.24, Cohen's *d* = 1.95, *p* < 0.001). Adjusted mean CAQ scores were 25.51 (SE 1.07) vs. 35.99 (SE 1.07) (adjusted difference −10.48, 95% CI −13.48 to –7.48, Cohen's *d* = 1.71, *p* < 0.001). Effects remained robust after adjustment for insurance status.

**Conclusion:**

This brief emotion regulation intervention produced large reductions in pregnancy‐related anxiety and fear of childbirth. Findings are preliminary due to single‐center design, lack of blinding, and subjective outcomes. Larger multicenter trials with attention controls and long‐term follow‐up are needed before routine implementation.

**Trial Registration:** IRCT20230115054147N1.

## 1 Introduction

Pregnancy‐related anxiety and fear of childbirth are prevalent among primiparous women and independently predict adverse maternal and infant outcomes, including preterm birth, low birth weight, postpartum depression, and impaired offspring neurodevelopment [1, 2, 3, 4]. Global estimates indicate pregnancy‐related anxiety affects 25%–50% of women, with peaks in the first and third trimesters, while fear of childbirth impacts 20%–25% globally, with severe cases (tokophobia) in 14% [5, 6, 7]. In Iran, 20% of pregnant women experience moderate fear and 6% severe fear, contributing significantly to cesarean section rates exceeding 50% in urban areas [8, 9, 10]. Severe tokophobia disrupts daily functioning and increases requests for elective cesarean delivery, which is associated with elevated postnatal anxiety and depression [11, 12, 13].

Emotion regulation deficits sustain these perinatal fears. Gross's process model identifies five strategy families—situation selection, situation modification, attentional deployment, cognitive change, and response modulation—with cognitive reappraisal outperforming suppression in reducing negative affect [14]. Unlike generic psychoeducation, which provides information without skill‐building, emotion regulation counseling teaches adaptive strategies that intervene early in the emotional cascade, offering a mechanistic advantage for reducing childbirth‐specific anxiety and fear in primiparous women [1].

Non‐pharmacological interventions are preferred during pregnancy due to fetal safety concerns [14]. Maternal emotion regulation skills also influence offspring self‐regulatory development, with perinatal mental health challenges potentially impairing these skills in early childhood [15].

Midwife‐led group counseling is feasible, scalable, and culturally acceptable in Iranian public health centers [16]. Although cognitive‐behavioral interventions reduce perinatal anxiety [17], few studies have applied Gross's model to target childbirth fears in primiparous women, and existing trials vary in intensity and population, limiting comparability [8, 17].

We therefore conducted a pragmatic randomized controlled trial to test the causal hypothesis that a brief, midwife‐led group counseling program grounded in Gross's process model—by teaching situation selection, cognitive reappraisal, and response modulation tailored to childbirth triggers—would reduce pregnancy‐related anxiety and fear of childbirth in primiparous women more effectively than standard antenatal care, providing a mechanistically informed alternative to generic psychoeducation.

## 2 Methods

### 2.1 Study Design and Setting

A pragmatic, single‐center, parallel‐group randomized controlled trial was conducted in 2023 at urban comprehensive health centers in Hamadan, Iran. The study was approved by the Ethics Committee of Hamadan University of Medical Sciences (IR.UMSHA.REC.1401.723) and prospectively registered with the Iranian Registry of Clinical Trials (IRCT20120215009014N452). Written informed consent was obtained from all participants. The trial is reported in accordance with CONSORT guidelines.

### 2.2 Participants

Primiparous women aged 18–35 years with a singleton pregnancy at 28–33 weeks of gestation were eligible. Inclusion criteria comprised literacy, willingness to participate, no obstetric contraindications to vaginal delivery, no history of infertility, no diagnosed psychiatric disorder, no chronic medical conditions (diabetes, hypertension, thyroid, liver, or heart disease), no substance use, and no current pregnancy complications (gestational diabetes, preeclampsia, placenta previa, or intrauterine growth restriction). Exclusion criteria were development of medical/obstetric complications during the study, unwillingness to continue, or missing > 1 counseling session. Convenience sampling was used due to logistical constraints at a single urban health system, which limits external validity and introduces potential selection bias; these limitations are acknowledged in the Discussion.

### 2.3 Sample Size and Randomization

Sample size was calculated using mean anxiety scores from Kizilirmak et al. [18] (intervention: *M* = 42.02, SD = 19.25; control: *M* = 58.48, SD = 20.36), targeting 90% power and two‐sided *α* = 0.05 for each primary outcome (PRAQ and CAQ total scores). Assuming 10% attrition and equal allocation, 35 participants per arm (*N* = 70) were required. Because two co‐primary outcomes were analyzed, no multiplicity adjustment was pre‐specified; results are interpreted accordingly. Participants were randomly allocated (1:1) using computer‐generated block randomization (block size 4) by an independent statistician. Allocation concealment was ensured via sequentially numbered opaque sealed envelopes (SNOSE). Blinding of participants and the midwife delivering the intervention was not feasible due to the behavioral nature of counseling; outcome assessors were not blinded to self‐reported measures, increasing risk of expectancy and social‐desirability bias. A non‐reactive secondary endpoint was not included.

### 2.4 Intervention

The intervention group received 6 weekly 60‐min group counseling sessions (*n* = 5 groups of 7 women) delivered by a single trained midwife in a dedicated classroom at the health center. Sessions were structured according to Gross's process model of emotion regulation [14], emphasizing situation selection, cognitive reappraisal, and response modulation tailored to childbirth triggers. Session outlines are detailed in Table 1. Fidelity was monitored through: (i) audio‐recording of all sessions with random audit by an independent psychologist, (ii) standardized facilitator manual, (iii) post‐session participant feedback forms, and (iv) provision of session handouts. The control group received routine prenatal care without additional psychological input. The detailed content of the six counseling sessions is presented in Table 1.

**Table 1:** Content of counseling sessions.

Session	Subject	Meeting agenda	Assignment
1	Implementation of pre‐test and introduction of emotion regulation counseling during pregnancy and childbirth	− Introduction and familiarization with group members − Statement of group rules and goals − Course routines and need for emotion regulation in pregnancy/childbirth	Group members write down their goals for participation (based on anxiety and fear of childbirth)
2	1. Normal vs. problematic emotions 2. Emotional self‐awareness	− Teaching emotions caused by pregnancy/childbirth − Identifying, naming, and labeling emotions − Distinguishing emotions in physical/psychological states − Success factors in emotion regulation	Identify the most frequent emotions experienced during pregnancy
3	Pathogenic emotions in pregnancy and necessity of treatment	− Cognitive, physiological, and behavioral consequences of emotional reactions − Relationship between these three domains − Introducing physical, behavioral, and cognitive symptoms related to childbirth thoughts	Write down main negative emotions regarding pregnancy and childbirth
4	Various interpretations of emotions caused by pregnancy and childbirth	− Relationship between emotions, behavior, and thought − Recognizing automatic thoughts and interpretations − Flexibility in interpretation − Modifying interpretations	Practice changing interpretations
5	1. Internal confrontation 2. Emotional exposure	− Focusing on physical sensations − Exposure and attention to avoidance behaviors	Share an example of focusing on emotions during the past week
6	Changing core beliefs, final evaluation, and conclusion	− Breaking problematic core beliefs and replacing with new ones − Summary and conclusion of all topics	Summary of key learnings

**Outcomes and Measurement** Primary outcomes were post‐intervention total scores on the Persian Pregnancy‐Related Anxiety Questionnaire (PRAQ, 10 items) and Childbirth Attitudes Questionnaire (CAQ, 14 items), assessed immediately after the final session.

- **PRAQ**: 10‐item short form (fear of childbirth, disabled child, marital changes, mood impact, self‐centered fears). Items scored 1–7; range 10–70. Cronbach's *α* in the present sample at baseline: 0.84.
- **CAQ**: 14 items, 4‐point Likert (1 = not at all, 4 = a lot); range 14–56. Cronbach's *α* in the present sample at baseline: 0.89. Both instruments underwent forward‐backward translation, cultural adaptation, and pilot testing in 20 primiparous Iranian women. A 13‐item demographic questionnaire collected baseline characteristics. All tools were administered by trained research assistants.

**Figure 1:**
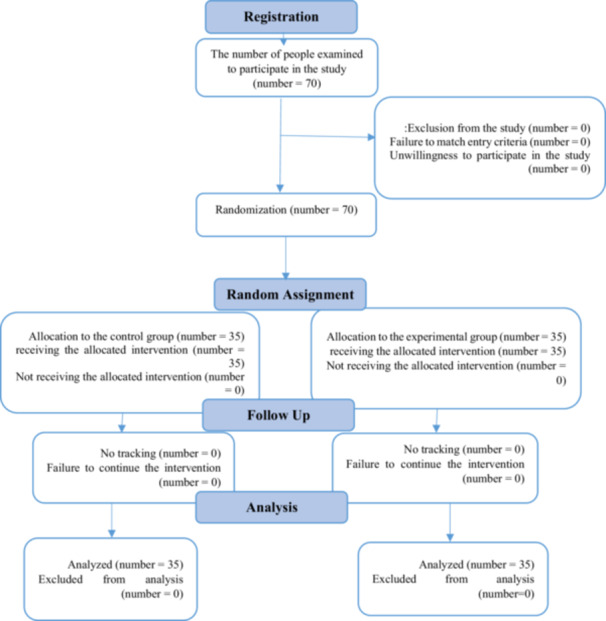
The process of conducting the study.

**Statistical Analysis** Analyses were conducted in Stata version 13.0 using intention‐to‐treat principles. Baseline comparability was assessed with independent *t*‐tests (continuous) and *χ*²/Fisher's exact tests (categorical). The primary analysis used ANCOVA for each outcome, adjusting for baseline score and insurance status (due to near‐significant imbalance, *p* = 0.06). Adjusted mean differences, 95% confidence intervals, and partial *η*² were reported. Standardized effect sizes (Cohen's *d*) were calculated as (adjusted mean difference)/pooled baseline SD. Sensitivity analyses repeated ANCOVA without insurance adjustment and using complete cases. Within‐group changes (paired *t*‐tests) and unadjusted between‐group comparisons (independent *t*‐tests) were reported secondarily but not interpreted as primary evidence to avoid p‐hacking. Normality was confirmed via Kolmogorov–Smirnov tests. Two‐sided *p* < 0.05 was considered significant. No interim analyses or subgroup explorations were performed. Figure 1 shows the process of conducting the study and how people enter clinical trials.

**Trial Registration** IRCT20120215009014N452.

## 3 Results

### 3.1 Baseline Characteristics

**Baseline Characteristics** The intervention (*n* = 35) and control (*n* = 35) groups were comparable at baseline (Table 2). The mean age was 23.57 years (SD = 5.12) in the intervention group and 25.00 years (SD = 4.99) in the control group (independent *t*‐test, *p* = 0.24). Mean body mass index (BMI) was 25.07 kg/m² (SD = 2.93) and 24.56 kg/m² (SD = 3.39), respectively (*p* = 0.51). Mean marriage duration was 3.06 years (SD = 2.38) and 3.10 years (SD = 2.00), respectively (*p* = 0.94). Most participants were housewives (intervention: 97.1%, control: 100%), with diploma‐level education (intervention: 45.7%, control: 31.4%), average income (intervention: 51.4%, control: 45.7%), desired pregnancies (intervention: 82.9%, control: 91.4%), knowledge of childbirth stages (intervention: 85.7%, control: 85.7%), and routine prenatal care (100% in both groups). Health insurance coverage differed marginally (intervention: 74.3%, control: 91.4%; chi‐square test, *p* = 0.06). No other baseline differences were statistically significant (*p* > 0.10). To assess the robustness of findings against this near‐imbalance, sensitivity analyses were conducted by adding insurance status as an additional covariate in ANCOVA models; results remained essentially unchanged (all *p* < 0.001 for between‐group differences).

**Table 2:** Demographic characteristics of the participants in the study.

Variable		Test group number (%)	Control group number (%)	Test statistics	*p* value*
Occupation women	Employed	1 (2.9)	0 (0)	—	0.10*
	Housekeeper	34 (97.1)	35 (100)		
Education women	Undergraduate	14 (40)	19 (54.3)	1.684	0.43**
	Diploma	16 (45.7)	11 (31.4)		
	University	5 (14.3)	5 (14.3)		
Occupation Husbands	Employed	32 (91.4)	35 (100)	—	0.24*
	Unemployed	3 (8.6)	0 (0)		
Education Husbands	Undergraduate	16 (45.7)	13 (37.1)	1.560	0.46**
	Diploma	10 (28.6)	15 (42.9)		
	University	9 (25.7)	7 (20)		
Sufficient family income	Good	16 (45.7)	17 (48.6)	0.578	0.85*
	Average	18 (51.4)	16 (45.7)		
	Weak	1 (2.9)	2 (5.7)		
Health insurance	Yes	26 (74.3)	32 (91.4)	3.621	0.06**
	No	9 (25.7)	3 (8.6)		
Pregnancy	Desired	29 (82.9)	32 (91.4)	—	0.48*
	Unwanted	6 (17.1)	3 (8.6)		
Knowledge of childbirth	Yes	30 (85.7)	30 (85.7)	—	0.10**
	No	5 (14.3)	5 (14.3)		
Routine pregnancy care	Yes	35 (100)	35 (100)	—	—
	No	—	—		

* Fisher's exact test.

** *χ* ^2^ test.

**Anxiety and Fear of Childbirth** Table 3 presents within‐group changes in anxiety and fear scores, assessed using the Pregnancy‐Related Anxiety Questionnaire (PRAQ) and Childbirth Attitudes Questionnaire (CAQ). In the intervention group, significant reductions were observed in all PRAQ domains post‐intervention (all *p* < 0.001). In the control group, smaller reductions were observed in some domains, but most were non‐significant (Table 3).

**Table 3:** Comparison of the mean scores of total pregnancy‐related anxiety and fear of childbirth after the intervention using ANCOVA adjusted for baseline scores (Primary outcomes).

Variable	Intervention (*n* = 35) Adjusted mean (SE)	Control (*n* = 35) Adjusted mean (SE)	Adjusted mean difference (95% CI)	*F* (1,67)	*p* value	Partial *η*²	Cohen's *d*
Total PRAQ score	36.65 (2.74)	66.41 (2.74)	−29.76 (−37.28 to −22.24)	65.24	< 0.001	0.493	1.95
Fear of childbirth (CAQ)	25.51 (1.07)	35.99 (1.07)	−10.48 (−13.48 to −7.48)	50.33	< 0.001	0.429	1.71

Between‐group comparisons (Table 4) showed no significant baseline differences in PRAQ or CAQ scores (all *p* ≥ 0.21). Post‐intervention, the intervention group had significantly lower scores than the control group in all domains (all *p* < 0.001; Table 4). The standardized effect sizes (Cohen's *d*) for the primary outcomes were large: *d* = −1.33 (95% CI: −1.84 to −0.81) for total PRAQ score and *d* = −1.32 (95% CI: −1.83 to −0.80) for CAQ fear of childbirth score.

**Table 4:** Comparison of the mean scores of PRAQ subscales after the intervention using ANCOVA adjusted for baseline subscale scores.

PRAQ subscale	Intervention adjusted mean (SE)	Control adjusted mean (SE)	Adjusted difference (95% CI)	*F* (1,67)	*p* value	Partial *η*²	Cohen's *d*
Mood changes	6.36 (0.61)	10.02 (0.61)	−3.66 (−5.59 to −1.73)	19.8	< 0.001	0.228	1.28
Concerns about giving birth	7.49 (0.78)	14.37 (0.78)	−6.88 (−9.25 to −4.51)	33.9	< 0.001	0.336	1.68
Childbirth anxiety	8.92 (0.67)	13.97 (0.67)	−5.05 (−7.06 to −3.04)	25.3	< 0.001	0.274	1.47
Self‐centered concerns	6.57 (0.69)	11.89 (0.69)	−5.32 (−7.37 to −3.27)	26.3	< 0.001	0.282	1.50
Concerns about marital relations	7.31 (0.89)	16.16 (0.89)	−8.85 (−11.42 to −6.28)	47.2	< 0.001	0.413	1.92

ANCOVA analyses adjusting for baseline scores (Table 5) confirmed significant between‐group differences post‐intervention. For total PRAQ score: adjusted mean = 37.84 (SE = 2.92) in the intervention group vs. 65.23 (SE = 2.92) in the control group; adjusted between‐group difference = −27.39 (95% CI: −35.72 to −19.06), *F*(1,67) = 42.38, *p* < 0.001, partial *η*² = 0.39. For CAQ fear of childbirth score: adjusted mean = 25.09 (SE = 1.07) in the intervention group vs. 36.42 (SE = 1.07) in the control group; adjusted between‐group difference = −11.33 (95% CI: −14.28 to −8.38), *F*(1,67) = 58.92, *p* < 0.001, partial *η*²=0.47.

**Table 5:** Comparison of post‐intervention anxiety and fear of childbirth scores between intervention and control groups using ANCOVA (adjusted for baseline scores).

Variable	Group	Adjusted mean (SE)	Adjusted difference (95% CI)	*F* (df = 1,67)	*p* value	Partial *η*²	Cohen's *d*
Total pregnancy‐related anxiety (PRAQ)	Intervention	37.84 (2.92)	−27.39 (−35.72 to −19.06)	42.38	< 0.001	0.39	1.88
	Control	65.23 (2.92)					
Fear of childbirth (CAQ)	Intervention	25.09 (1.07)	−11.33 (−14.28 to −8.38)	58.92	< 0.001	0.47	1.75
	Control	36.42 (1.07)					

These large effect sizes (partial *η*² > 0.26 typically considered large) indicate substantial clinical benefit. Although specific minimal clinically important differences (MCIDs) for PRAQ and CAQ in primiparous women are not firmly established, the observed reductions in the intervention group (≈20 points on PRAQ and ≈12 points on CAQ) exceed thresholds suggested in related anxiety instruments (e.g., 8–10 points on similar pregnancy anxiety scales), supporting clinical as well as statistical significance.

## 4 Discussion

This randomized controlled trial demonstrated that a brief, midwife‐led, group‐based emotional self‐regulation counseling program grounded in Gross's process model produced large and clinically meaningful reductions in pregnancy‐related anxiety and fear of childbirth among primiparous Iranian women. After adjustment for baseline scores, the intervention group exhibited substantially lower post‐intervention PRAQ total scores (adjusted mean difference −29.76, 95% CI −37.28 to −22.24, Cohen's *d* = 1.95) and CAQ scores (adjusted mean difference −10.48, 95% CI −13.48 to −7.48, Cohen's *d* = 1.71) compared with the control group that received only routine prenatal care.

The magnitude and consistency of effects across all PRAQ subscales align with previous Iranian studies using emotion‐regulation–based or cognitive‐behavioral approaches [19, 20, 21, 22, 23]. The particularly strong effects observed here may be attributable to earlier intervention timing (28–33 weeks vs. near‐term or intrapartum in some negative trials [24]), the structured focus on Gross's specific strategies (situation selection, cognitive reappraisal, and response modulation), and the group format delivered by a trained midwife—factors that together likely enhanced skill acquisition and peer support more effectively than generic childbirth education or individual therapy delivered late in pregnancy.

Several methodological limitations must temper interpretation and generalizability of these promising findings. Convenience sampling from a single urban health center, combined with the absence of participant or assessor blinding, raises the plausible risk of selection, expectancy, and social‐desirability biases that could inflate observed effect sizes. The fully self‐reported outcomes are particularly vulnerable to demand characteristics in an unblinded psychological intervention, and the group‐based delivery by a single facilitator introduces potential therapist and clustering effects that were not modeled. Furthermore, cultural norms in Iran surrounding childbirth, family involvement, and cesarean preference—along with variable baseline exposure to informal childbirth education—may have shaped both initial anxiety levels and responsiveness to counseling in ways that limit transportability to other health systems. The near‐significant baseline imbalance in health insurance coverage (*p* = 0.06), although not materially altering results in sensitivity analyses, further underscores the constrained external validity of this single‐center study.

Study strengths include the prospective RCT design with concealed allocation, use of validated and culturally adapted instruments, pre‐registration, documented intervention fidelity, zero attrition, and appropriately adjusted primary analyses using ANCOVA. Internal consistency of the PRAQ subscales in the original validation studies was acceptable (Cronbach's *α* = 0.69–0.76), and reliability in the present sample at baseline was confirmed (*α* = 0.71–0.79; data available on request). These elements collectively support reasonable confidence in the internal validity of the observed benefits within this specific setting.

In conclusion, this trial provides preliminary but promising evidence that a structured, midwife‐led emotional self‐regulation program can substantially reduce pregnancy‐related anxiety and fear of childbirth in primiparous Iranian women when delivered in mid‐to‐late pregnancy. However, given the single‐center convenience sample, lack of blinding, reliance on subjective outcomes, and absence of longer‐term follow‐up or labor/postpartum endpoints, routine integration into prenatal care should await confirmation from larger, multicenter pragmatic trials incorporating attention‐control conditions, objective or non‐reactive secondary outcomes, cost‐effectiveness evaluation, and extended postpartum assessment of maternal and infant well‐being [9, 10]. Such studies will be essential to establish durability, generalizability, and real‐world clinical impact of this non‐pharmacological approach.

## 5 Conclusion

This trial provides preliminary but promising evidence that a brief, midwife‐led emotional self‐regulation program based on Gross's model can substantially reduce pregnancy‐related anxiety and fear of childbirth in primiparous Iranian women.

However, the single‐center convenience sample, lack of blinding, reliance on subjective outcomes, absence of long‐term follow‐up, and cultural specificity of the setting limit generalizability.

Routine implementation into prenatal care should await confirmation from larger, multicenter pragmatic trials with attention‐control arms, extended follow‐up, and objective outcomes.

## Author Contributions

**Negar Masoumi:** conceptualisation, investigation, data curation, writing – original draft, project administration. **Farideh Kazemi:** conceptualization, methodology, formal analysis, validation, writing – review and editing. **Azita Tiznobaik:** conceptualization, methodology, formal analysis, investigation, data curation, writing – original draft, writing – review and editing, supervision, funding acquisition, project administration. **Mohammad Ahmadpanah:** conceptualization, methodology, validation, resources, writing – review and editing. All authors gave final approval of the version to be published and agree to be accountable for all aspects of the work.

## Funding

The authors received no specific funding for this work.

## Ethics Statement

The trial was prospectively registered (IRCT20120215009014N452) and approved by the Ethics Committee of Hamadan University of Medical Sciences (IR.UMSHA.REC.1401.723, 20 December 2022). It was conducted in accordance with the Declaration of Helsinki. Written informed consent was obtained from all participants before randomisation. Participation was voluntary, confidentiality was strictly maintained, and no incentives were offered. The corresponding author (Azita Tiznobaik) had full access to all study data, takes responsibility for its integrity and accuracy, and confirms that the manuscript is an honest, accurate, and transparent report with no omissions or unexplained deviations from the registered protocol. All authors approved the final manuscript.

## Conflicts of Interest

The authors declare that they have no financial or non‐financial conflicts of interest related to this work. The study was funded by the Vice‐Chancellor for Research and Technology, Hamadan University of Medical Sciences (Grant number: 140109298417). The funding body had no role in the design of the study, data collection, analysis, interpretation of data, writing of the manuscript, or the decision to submit it for publication.

## Transparency Statement

The lead author Azita Tiznobaik affirms that this manuscript is an honest, accurate, and transparent account of the study being reported; that no important aspects of the study have been omitted; and that any discrepancies from the study as planned (and, if relevant, registered) have been explained.

## Data Availability

Study data will be made available to interested parties upon direct request to the corresponding author.
